# Gold Nanocluster-Based Fluorometric Banoxantrone Assay Enabled by Photoinduced Electron Transfer

**DOI:** 10.3390/nano12111861

**Published:** 2022-05-30

**Authors:** Kai-Yuan Huang, Wen-Hui Weng, Xin Huang, Hong-Xiang Huang, Hamada A. A. Noreldeen, Hao-Hua Deng, Wei Chen

**Affiliations:** 1 Fujian Key Laboratory of Drug Target Discovery and Structural and Functional Research, School of Pharmacy, Fujian Medical University, Fuzhou 350004, China; 15980269577@163.com (K.-Y.H.); wwh18344910886@163.com (W.-H.W.); xinhuang5649@163.com (X.H.); hhx1830400660@163.com (H.-X.H.); haali8651@gmail.com (H.A.A.N.); 2 Department of Pharmacy, Fujian Provincial Hospital, Fuzhou 350001, China

**Keywords:** gold nanocluster, banoxantrone, photoinduced electron transfer, photoluminescence quenching, Coulomb interaction

## Abstract

Monitoring the blood concentration of banoxantrone (AQ4N) is important to evaluate the therapeutic efficacy and side effects of this new anticancer prodrug during its clinical applications. Herein, we report a fluorescence method for AQ4N detection through the modulation of the molecule-like photoinduced electron transfer (PET) behavior of gold nanoclusters (AuNCs). AQ4N can electrostatically bind to the surface of carboxylated chitosan (CC) and dithiothreitol (DTT) co-stabilized AuNCs and quench their fluorescence via a Coulomb interaction-accelerated PET process. Under optimized experimental conditions, the linear range of AQ4N is from 25 to 200 nM and the limit of detection is as low as 5 nM. In addition, this assay is confirmed to be reliable based on its successful use in AQ4N determination in mouse plasma samples. This work offers an effective strategy for AQ4N sensing based on fluorescent AuNCs and widens the application of AuNCs in clinical diagnosis and pharmaceutical analysis.

## 1. Introduction

Banoxantrone (AQ4N) is a novel hypoxia-activated prodrug that exhibits anticancer activity following bioreduction by heme-containing reductases to AQ4, an effective DNA intercalator and topoisomerase II inhibitor [1]. Although preclinical trials have revealed that AQ4N is relatively nontoxic in tumor treatments, the overdose use of AQ4N still faces the risk of severe side effects including cardiotoxicity and myelosuppression [2,3,4]. Consequently, the development of methods for precise monitoring and quantification of the blood concentration of AQ4N is of great significance. So far, high-performance liquid chromatography (HPLC) [5] and mass spectrometry (MS) [6] have been utilized for the detection of AQ4N. However, these approaches may suffer from some drawbacks such as time consumption and the involvement of toxic solvents. Therefore, it is greatly welcomed to establish rapid, accurate, and sensitive analytical techniques for measuring AQ4N concentration.

Photoinduced electron transfer (PET), where an excited electron transfers from an electron donor to an electron acceptor, is one of most popular mechanisms in the design of fluorescence-sensing strategies [7,8]. The PET process can happen within several picoseconds, which will quickly deactivate the emission-excited state of fluorophores and finally cause fast and remarkable luminescence quenching [9,10]. According to a classical theory, the dynamics of PET can be easily regulated by the excited-state parameters of electron transfer reactants including driving force (ΔG), reorganization energy (λ), and electron coupling factor (HDA) [11]. Such features make PET an efficient technique to construct powerful chemo/biosensors for fluorescence detection of DNA [12,13], proteins [14,15], small biomolecules [16,17], drugs [18,19], ions [20,21], and so forth.

**Scheme 1:**
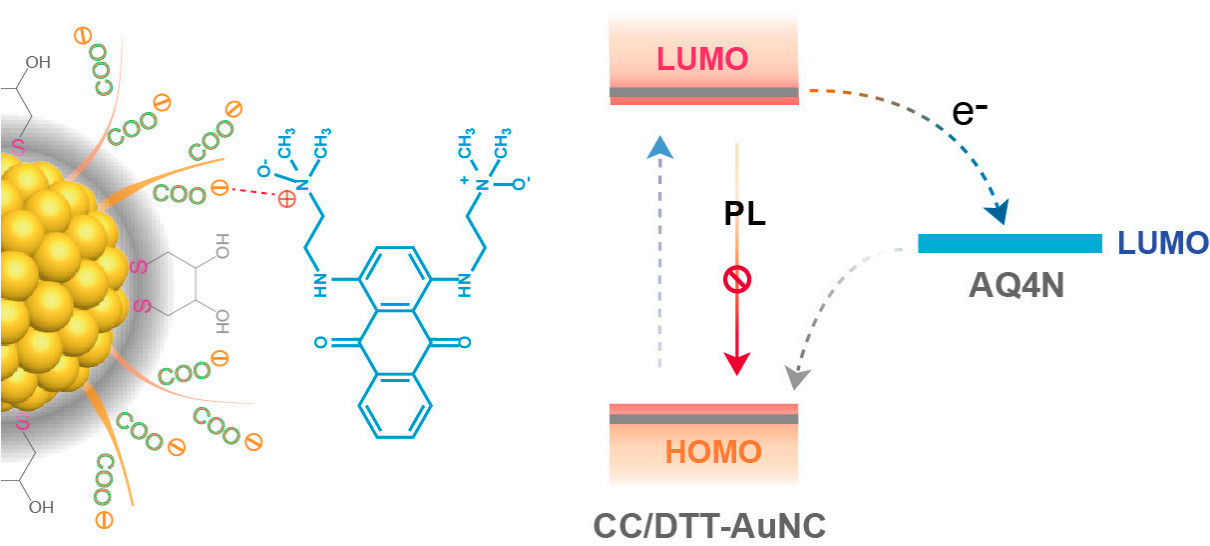
Illustration of the design principle of this AQ4N sensing strategy.

Ultrasmall gold nanoparticles (commonly known as gold nanoclusters, AuNCs) with a metallic core diameter less than 2 nm have become a new kind of fluorescent nanomaterial for developing PET sensors owing to their intriguing properties, such as long-wavelength photoluminescence (PL) and distinguishable electrochemical performance [22,23,24,25,26,27]. In a typical PET process of AuNCs, relatively weak electron–hole coupling will suppress Auger-assisted electron transfer behavior and thereby give rise to a distinguished Marcus inverted region [28,29,30,31]. Moreover, the fluorescence of a AuNC-based PET probe is sensitive to the alteration of their physicochemical characteristics, for instance, the surface charge, redox state, metallic core size, and ligand length [32,33,34,35]. Inspired by these attractive properties mentioned above, herein, a new fluorometric AQ4N assay was rationally designed by triggering molecule-like PET behavior of AuNCs (Scheme 1), in which the carboxylated chitosan (CC) and dithiothreitol (DTT) co-stabilized AuNCs (CC/DTT-AuNCs) served as an electron donor and AQ4N acted as an electron acceptor. Positively charged AQ4N can attach to the negatively charged CC/DTT-AuNCs. After that, AQ4N accepts an electron from the photoexcited CC/DTT-AuNCs and suppresses the PL as a result. The PET process between AQ4N and CC/DTT-AuNCs was systematically investigated and was solidly supported by experimental and calculation results. Furthermore, the proposed method was also applied to monitor AQ4N concentration in mouse plasma with satisfactory results.

## 2. Materials and Methods

### 2.1. Chemicals and Reagents

AQ4N, HAuCl_4_·3H_2_O, cisplatin (DDP), histidine (His), cyclophosphamide (CAR), L-cysteine (Cys), L-glutathione (GSH), ascorbic acid (AA), carboxylated chitosan (CC), dithiothreitol (DTT), bovine serum albumin (BSA), and N-acetyl-L-cysteine (NAC) were purchased from the Aladdin Reagent Co. (Shanghai, China) (http://www.aladdin-e.com/, accessed on 12 January 2022) Other analytical-grade chemicals were commercial and used directly without any further purification. Ultrapure water (>18 kΩ) was used throughout the experiments.

### 2.2. Preparation of CC/DTT-AuNCs

The AuNCs employed in this investigation were prepared and purified following our previously reported approach [30]. The optical properties of the resulting CC/DTT-AuNCs can be preserved without any change for at least three months at 4 °C in a refrigerator.

### 2.3. AQ4N Determination

AQ4N detection was performed using the following procedure: 0.1 mL of CC/DTT-AuNC solution was added to 1.9 mL of standard AQ4N solutions with different concentrations (in 20 mM phosphate buffer, pH 6.0). The emission spectra of the mixtures were monitored directly at an excitation wavelength of 290 nm.

### 2.4. AQ4N Extraction and Quantification

The extractions of AQ4N were carried out as follows: three adult mice each were given a dosage of 0.1 mL/g AQ4N solution (10 mM) by intraperitoneal injection. At the time point of 15 min, the whole blood was taken from orbits, placed into heparinized tubes on ice, centrifugated at 3000 rpm for 5 min, and then the separated plasma was collected. Finally, 100 μL of the obtained plasma was mixed with 300 μL of methanol followed by centrifugation at 7000 rpm for 8 min, and the supernatant was used in subsequent experiments.

Measurement of the AQ4N content in the extractions was performed by our developed method: 15 μL of the supernatant and 0.1 mL of CC/DTT-AuNC solution were introduced into 1.885 mL phosphate buffer solution (20 mM, pH 6.0). Then, the emission spectra of resulting solutions were recorded immediately with an excitation wavelength of 290 nm.

Quantification of AQ4N concentration in the extractions by HPLC: A Shimadzu LC-15C (Shimadzu, Japan), equipped with an Agilent Zorbax SB-C18 column (5 μm, 46 × 250 mm i.d.) (Agilent Technology, Palo Alto, CA, USA) was operated at 25 °C. The separation conditions were: 1‰ formic acid solution containing 10 mM ammonium formate: acetonitrile, 75:25; a flow rate of 1 mL/min; and an injected volume of 20 µL for the supernatant. A 245 nm SPD UV–Vis detector was used.

### 2.5. Computational Methodology

The luminescence quenching efficiency (E%) was determined using the following equation:

(1) $$E\%=(1-\frac{F}{F_{0}})\;\times \;100\%$$

where F_0_ and F are luminescence intensities of CC/DTT-AuNCs at 645 nm in the absence and presence of AQ4N, respectively.

The inner filter effect (IFE) was corrected by the following equation [36]:

(2) $$\mathrm{CF}=\frac{F_{\mathrm{cor}}}{F_{\mathrm{obsd}}}=\frac{2.3{\mathrm{dA}}_{\mathrm{ex}}}{1-10^{-{\mathrm{dA}}_{\mathrm{ex}}}}10^{{\mathrm{gA}}_{\mathrm{em}}}\frac{2.3{\mathrm{sA}}_{\mathrm{em}}}{1-10^{-{\mathrm{sA}}_{\mathrm{em}}}}$$

where F_obsd_ is the PL intensity of CC/DTT-AuNCs at 645 nm and Fcor is the corrected PL intensity obtained by removal of the IFE from Fobsd, while A_ex_ and A_em_ are the absorbances recorded at 290 nm (excitation wavelength) and 645 nm (emission wavelength), respectively. s, d, g are instrument-related geometrical parameters that are usually fixed at 0.1, 0.4, and 1.0 cm, respectively. CF represents a believable correction factor, which should not exceed 3.

The driving force was calculated by the Rehm–Weller equation [37]:

(3) $$\Delta G=e(E_{\mathrm{ox}}^{D}\;-\;E_{\mathrm{red}}^{A})-E_{0,0}+C$$

where $E_{\mathrm{ox}}^{D}$ (1.24 V, Figure S1) and $E_{0,0}$ (2.65 eV) are the first oxidation potential and 0–0 singlet–singlet excitation energy of CC/DTT-AuNCs, respectively; $E_{\mathrm{red}}^{A}$ is the first reduction potential of AQ4N (–0.68V, Figure S2); and C represents the solvent-dependent Coulombic interaction energy, which is commonly ignored in water.

### 2.6. Instruments

Optical absorption spectra were conducted on a Shimadzu UV-2450 spectrophotometer (Shimadzu, Japan). A Cary Eclipse fluorescence spectrophotometer (Agilent, Palo Alto, CA, USA) was used to measure the fluorescence spectra. The transmission electron microscopy (TEM) image on a JEM-2100 microscope (JEOL, Tokyo, Japan) was employed to characterize the shape and size of CC/DTT-AuNCs. An F900 microsecond time-correlated single-photon-counting (TSPC) fluorescence lifetime spectrometer (Edinburgh Analytical Instruments, Edinburgh, UK) was used to determine the PL lifetime changes of CC/DTT-AuNC solutions in the absence and presence of AQ4N. The first redox potentials of the PET reactants were measured in a three-electrode system (a platinum wire, a bare glassy carbon electrode, and an Ag/AgCl electrode were used as counter electrode, working electrode, and reference electrode, respectively) performed on a CHI 660C electrochemical analyzer (CHI Instrument Inc., Austin, TX, USA).

## 3. Results and Discussion

### 3.1. Designing Strategy

**Figure 1:**
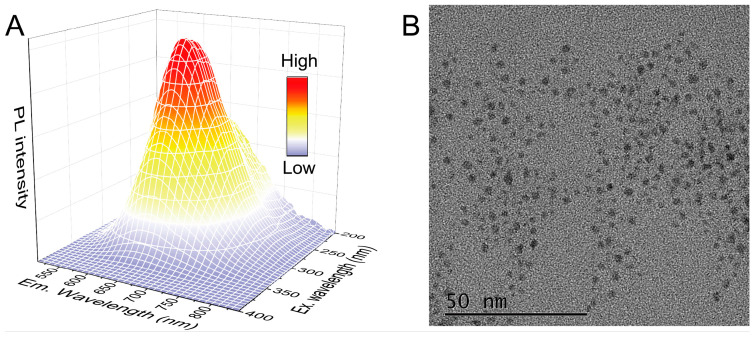
(**A**) Three-dimensional PL spectra of the prepared CC/DTT-AuNCs. (**B**) TEM image of CC/DTT-AuNCs.

To rationally construct the fluorescence AQ4N sensor, we first screened the potential AuNC nanoprobe in light of the PET theory and the physicochemical characteristics of AQ4N (which is an electron-deficient molecule with a positive surface charge [38]). The theorical investigations have pointed out that a high quenching ratio of fluorescent PET sensors will appear when the driving force approaches the reorganization energy [11]. Additionally, a previous report has demonstrated that a long excited-state lifetime will be of benefit for improving the sensitivity of luminescent PET sensors [39]. Recently, it was also found that the Coulomb interaction of ground-state PET reactants is capable of promoting fluorescence quenching efficiency due to an accelerated diffusion rate [40]. Taken together with these principles, CC/DTT-AuNCs were selected as the fluorescent nanoprobe, owing to their molecule-like PET behavior, appropriate oxidation potential, long luminescence lifetime, and negative surface charge (Table S1) [30,31]. The obtained CC/DTT-AuNCs exhibited two weak single-electron transitions in their UV–vis absorption spectrum, rather than the surface plasmons observed in metallic-state gold nanoparticles (Figure S3). The 3D PL spectra analysis of these AuNCs showed that the excitation and emission bands were centered at 290 and 645 nm, respectively (Figure 1A). A transmission electron microscope (TEM) image revealed that the spherical CC/DTT-AuNC particles were well-dispersed with a mean diameter of about 2.0 nm (Figure 1B).

### 3.2. Luminescence Quenching Mechanism Study

**Figure 2:**
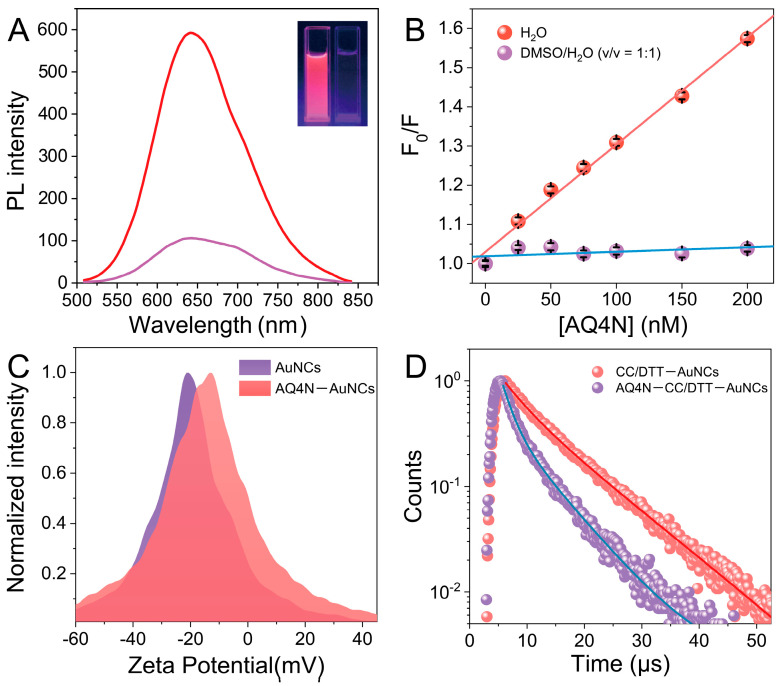
(**A**) The photoemission spectra of CC/DTT-AuNCs (red line) and CC/DTT-AuNCs + 1 μM AQ4N (blue line). Inset shows the photographs of CC/DTT-AuNC solutions without (left) and with (right) the introduction of 1 μM AQ4N under a 254 nm UV light. (**B**) The effect of solvent polarity on the luminescence suppression of AQ4N-CC/DTT-AuNCs system. (**C**) Zeta potentials of CC/DTT-AuNCs in the absence and presence of 10 μM AQ4N. (**D**) Emission decay profiles of CC/DTT-AuNCs and CC/DTT-AuNCs + 1 μM AQ4N.

When an aqueous solution of AQ4N (1 μM) was introduced into CC/DTT-AuNCs, a remarkable luminescence suppression was observed (Figure 2A). To better understand the luminescence quenching mechanism, a series of experiments were performed. IFE can be ruled out because the luminescence quenching efficiencies are almost identical before and after the removal of IFE by a correction formula (Figures S4 and S5, Table S2). Owing to the strong solvent-dependent quenching (Figure 2B), we can further safely exclude a Förster resonance energy transfer (FRET) mechanism since the appearance of FRET will not generate a polarity-sensitive electron transfer state [41]. Our previous works have demonstrated that the luminescence quenching in AuNC-cationic molecule assemblies is originated from a Coulomb interaction-accelerated PET process [30,31]. The possibility of this effect in the present system was then discussed. The Coulomb interaction between CC/DTT-AuNCs and AQ4N can be verified by the declined surface potential of CC/DTT-AuNCs after the addition of AQ4N (Figure 2C). By using the Rehm−Weller equation, the driving force of the AQ4N-CC/DTT-AuNCs PET system was calculated to be 0.74 eV, which is close to the theoretical value of reorganization energy (0.67 eV, Table S1) and agrees well with the observed obvious luminescence quenching. Moreover, an apparent decrease in the average fluorescence lifetime of CC/DTT-AuNCs (from 8.9 to 1.7 μs) upon the addition of AQ4N suggested that an additional nonradiative relaxation pathway is activated by the formation of an electron-transfer state (Figure 2D) [42,43]. These data firmly proved that the Coulomb interaction-accelerated PET is the dominant mechanism for the AQ4N-induced PL quenching of CC/DTT-AuNCs.

### 3.3. Optimizing the Test Conditions for AQ4N

**Figure 3:**
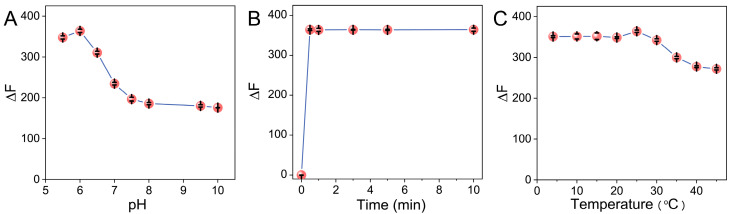
Influences of (**A**) solution pH, (**B**) incubation time, and (**C**) incubation temperature on the proposed AQ4N sensing system. The concentration of AQ4N is 0.5 μM.

The main operational parameters, including the solution pH, incubation time, and incubation temperature for the CC/DTT-AuNC-based AQ4N luminescent sensing system, were examined. The value of ΔF (ΔF = F_0_ − F, where F_0_ and F are the luminescence intensities of CC/DTT-AuNCs at 645 nm before and after the addition of AQ4N, respectively) was calculated and used to evaluate the quenching ability of AQ4N. The influence of solution pH on the AQ4N detection assay was tested by varying the phosphate buffer pH from 5.5 to 10, and the results displayed that the maximum ΔF appeared at pH 6.0 (Figure 3A). Therefore, a pH 6.0 solution was chosen in the subsequent experiments. The time-dependent curve manifested that the luminescence response of CC/DTT-AuNCs toward AQ4N is quite rapid, which can be finished within 30 s (Figure 3B). Hence, PL intensities of the reaction solutions were recorded immediately. Finally, we tested the influence of the incubation temperature on the AQ4N sensing system, and the results showed that ΔF reaches a maximum value at 25 °C and decreases with a further increase in temperature. The decreasing ΔF value at higher temperatures is reasonably owed to the weak electronic wavefunction coupling of the reactants according to a classical theory [11]. Thus, we conducted the follow-up experiments at 25 °C.

### 3.4. Sensing Performance

**Figure 4:**
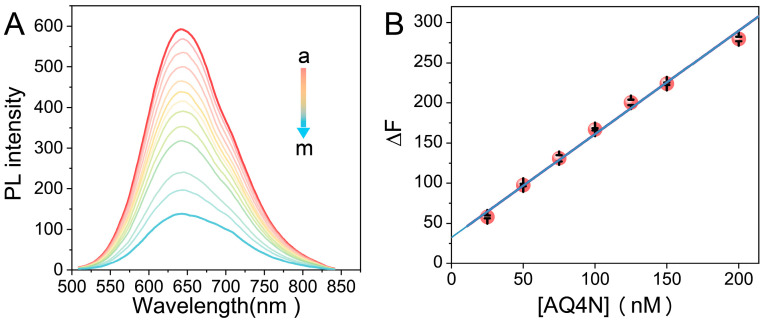
(**A**) The photoemission spectra of CC/DTT-AuNCs in the presence of various amounts of AQ4N. From a to m: 0, 25, 50, 75, 100, 125, 150, 175, 200, 250, 400, 500, and 750 nM. (**B**) ΔF as a function of AQ4N concentration.

To ensure that this method will exhibit satisfied sensitivity, the optimal conditions (pH 6.0 and 25 °C) were employed to assess the performance of a CC/DTT-AuNC-based PET sensor toward AQ4N detection. As displayed in Figure 4A, the PL intensity of the CC/DTT-AuNCs decreased gradually with increasing AQ4N concentrations. A good linear relationship (ΔF = 1.3[AQ4N] + 32.4; [AQ4N]: nM; r = 0.997) emerges between the ΔF value and the AQ4N concentration over the range of 25–200 nM (Figure 4B). The limit of detection (LOD) was calculated to be as low as 5 nM (based on 3σ/S, where σ and S are the standard deviation of blank signals and slope of the working curve, respectively), which is obviously lower than the reported HPLC method [5]. A 1.7% relative standard deviation (RSD) was obtained for six parallel tests ([AQ4N] = 150 nM), suggesting the high reproducibility of the proposed assay system.

**Figure 5:**
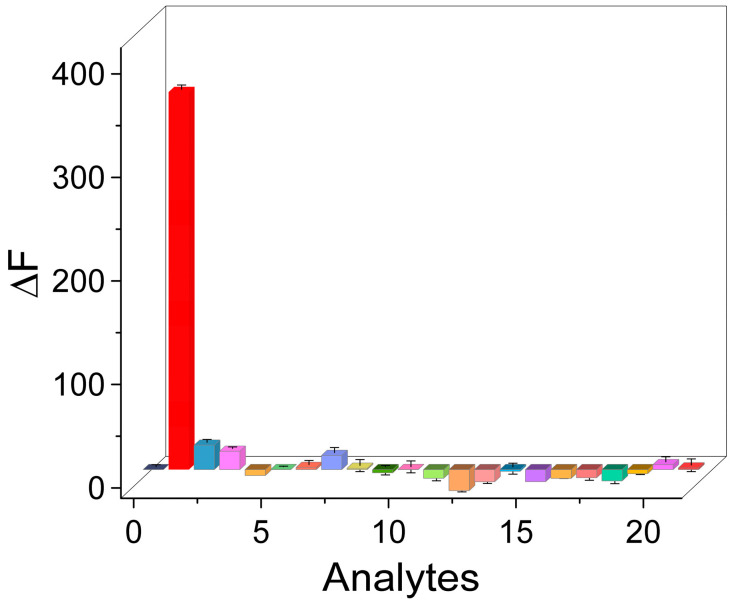
Specificity of the developed detection method for AQ4N. The labels of number marked on the X-axis correspond to (0) blank, (1) AQ4N, (2) Fe^2+^, (3) Fe^3+^, (4) Zn^2+^, (5) Mg^2+^, (6) K^+^, (7) Na^+^, (8) HCO_3_^−^, (9) GSH, (10) His, (11) BSA, (12) Ca^2+^, (13) NAC, (14) CAR, (15) AA, (16) glucose, (17) urea, (18) Cys, (19) DDP, (20) alizarin, and (21) rhein. The concentrations of AQ4N and BSA were set at 0.5 μM and 40 μg/mL, respectively. The concentrations of alizarin and rhein were 1 μM. The concentrations of other tested substances were 10 μM.

To validate the specificity of the developed method, potential interfering agents were added to this AQ4N determination system. It can be clearly seen from Figure 5 that only AQ4N can induce a notable change for CC/DTT-AuNC luminescence, demonstrating the high specificity of this approach.

### 3.5. Detection of AQ4N in Real Samples

Due to the dose-related toxicity of AQ4N, it is necessary to monitor the blood AQ4N concentration during clinical applications. The feasibility of the constructed PET sensor was testified by analyzing the concentration of AQ4N in mouse plasma samples. The detection results were summarized in Table 1, wherein the AQ4N concentrations were determined by our proposed approach and the HPLC method, respectively. A *t*-test and an F-test conducted at a confidence level of 95% revealed that there were no significant differences in the values measured by using the two methods. These results confirmed that this fluorometric assay is appropriate for the quantification of AQ4N in real samples.

## 4. Conclusions

In summary, relying on the molecule-like PET property of AuNCs, a new fluorescence-sensing strategy was developed for rapid, sensitive, and selective monitoring of AQ4N. Based on the results of energetic and optical studies, it was found that the significant luminescence quenching in AQ4N-CC/DTT-AuNCs complexes mainly stems from a Coulomb interaction-accelerated PET process. Compared with the frequently used HPLC method, the strategy demonstrated here not only possesses comparable reproducibility and feasibility but also averts the complicated processing steps and use of toxic organic solvents. We believe that this paper will promote further works on the fabrication and application of fluorescence PET sensors in drug discovery and clinical practice.

## Figures and Tables

**Table 1:** Detection of AQ4N in mouse plasma.

Sample	Our method (μM, Mean ± SD, *n* = 3)	HPLC Method (μM, mean ± SD, *n* = 3)	F-Test ^a^	*t*-Test ^a^
1	82.5 ± 1.2	78.2 ± 0.02	7.93	1.42
2	81.8 ± 0.6	78.3 ± 0.04	5.23	1.79
3	74.6 ± 1.9	78.4 ± 0.08	5.87	1.18

^a^ F_0.05,2,2_ =19.00, t_0.05,4_ = 2.776.

## Data Availability

The data presented in this study are available on request from the corresponding author.

## Supplementary Materials

The following supporting information can be downloaded at: https://www.mdpi.com/article/10.3390/nano12111861/s1, Figure S1: Cyclic voltammogram curve of CC/DTT-AuNCs; Figure S2: Cyclic voltammogram curve of AQ4N; Figure S3: UV–vis absorption spectrum of CC/DTT-AuNCs; Figure S4: The absorption spectrum of AQ4N; Figure S5: The AQ4N concentration-dependent quenching efficiency determined for the CC/DTT-AuNCs before and after correction by the IFE formula; Table S1: Screening of AuNCs candidate using PET theory; Table S2: IFE on the luminescence response of CC/DTT-AuNCs toward AQ4N. References [30,44,45,46,47,48,49,50,51] were cited in supplementary materiasl.
