# Supplementary material for: Gold Nanocluster-Based Fluorometric Banoxantrone Assay Enabled by Photoinduced Electron Transfer
Source: Nanomaterials (Basel). 2022 May 30;12(11):1861. doi: 10.3390/nano12111861 (PMC9182391; doi:10.3390/nano12111861)
Supplement: Supplementary file 1 [file nanomaterials-12-01861-s001.zip › nanomaterials-1717492-supplementary.pdf]

Supplementary Materials

Gold Nanocluster-Based Fluorometric Banoxantrone Assay Enabled by Photoinduced Electron Transfer

Kai-Yuan Huang [1,†], Wen-Hui Weng [1,2,†], Xin Huang [1], Hong-Xiang Huang [1], Hamada A. A. Noreldeen [1], Hao-Hua Deng [1,*] and Wei Chen [1,*]

[1] Fujian Key Laboratory of Drug Target Discovery and Structural and Functional Research,
School of Pharmacy, Fujian Medical University, Fuzhou 350004, China; 15980269577@163.com (K.-Y.H.); wwh18344910886@163.com (W.-H.W.); xinhuang5649@163.com (X.H.); hhx1830400660@163.com (H.-X.H.); haali8651@gmail.com (H.A.A.N.)

[2] Department of Pharmacy, Fujian Provincial Hospital, Fuzhou 350001, China

* Correspondence: hhdeng@fjmu.edu.cn (H.-H.D.); chenandhu@163.com (W.C.)

† These authors contributed equally to this work.

Calculation of reorganization energy (λ)

The values of λ were calculated as follows [1]:

$$\lambda = \lambda_i + \lambda_o \approx \lambda_o$$

$$\lambda_o = \frac{e^2}{4\pi\varepsilon_o}\left(\frac{1}{2r_D} + \frac{1}{2r_A} - \frac{1}{r_{DA}}\right)\left(\frac{1}{n^2} - \frac{1}{\varepsilon}\right)$$

$$r_{DA} = r_D + r_A$$

in which, the λ_i is inner reorganization energy and λ_o is the outer reorganization energy; the ε_o r_D, r_A, r_{DA}, n, and ε are the permittivity of vacuum (8.85×10^{-12} Fm^{-1}), the radius of the gold nanoclusters (AuNCs) and banoxantrone (AQ4N), the center-center distance between the AuNCs and the AQ4N, the refractive index (1.33 for water), and the static dielectric constant (78.5 for water) of the solvent, respectively. In the present case, radius of AuNCs were obtained from TEM image or previously reported data [2-9]. The radius of AQ4N was 5 Å, which was obtained from theoretical calculations.

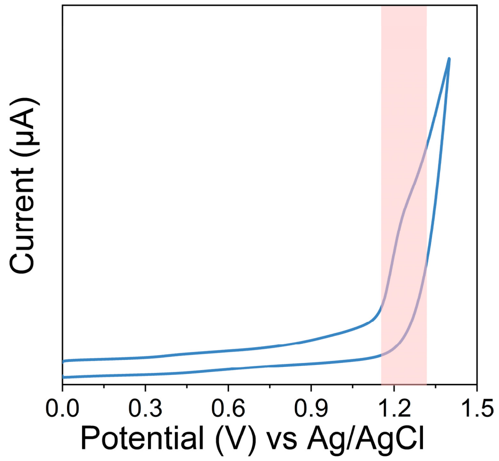

Figure S1. Cyclic voltammogram curve of CC/DTT-AuNCs in 0.1 M KCl solution (pH 6.0, scan rate: 50 mV/s).

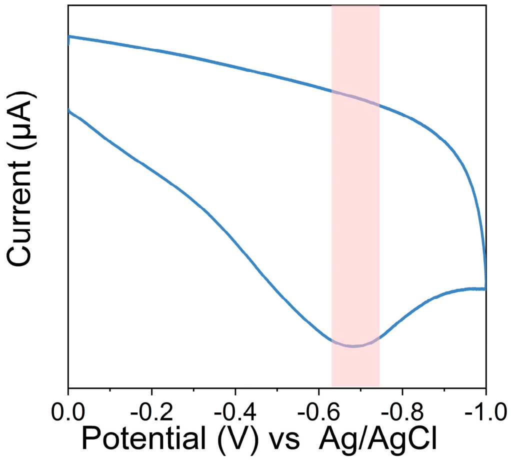

Figure S2. Cyclic voltammogram curve of AQ4N in 0.1 M KCl solution (scan rate: 50 mV/s).

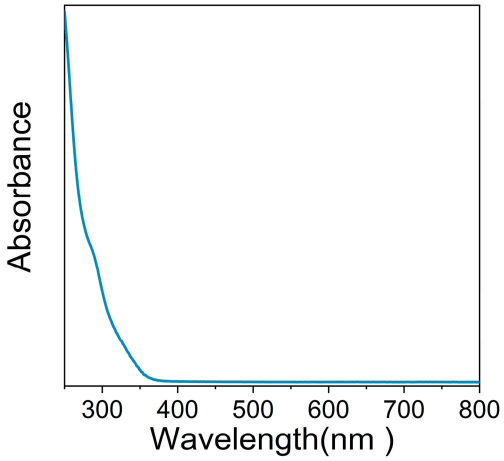

Figure S3. UV-vis absorption spectrum of CC/DTT-AuNCs.

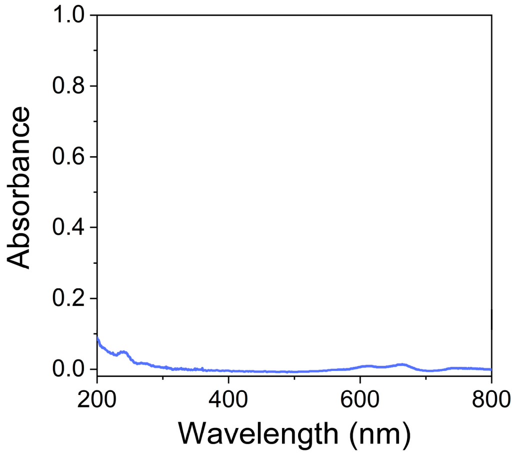

Figure S4. The absorption spectrum of AQ4N (1 μM).

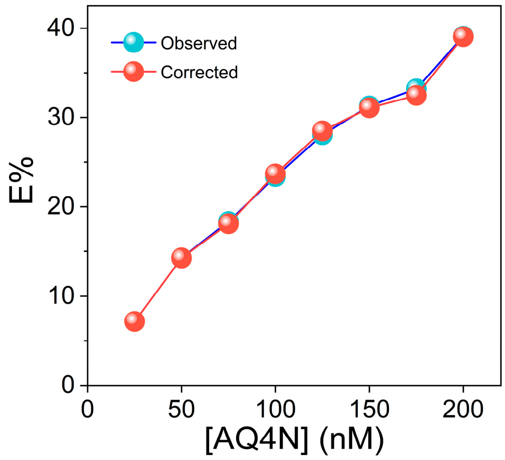

Figure S5. The AQ4N concentration-dependent quenching efficiency determined for the CC/DTT-AuNCs before and after correction by the IFE formula.

Table S1. Screening of AuNCs candidate using PET theory.

Probe	E_{ox} (V)[a]	τ (μs)[b]	QY (%)[c]	- ΔG (eV)[d]	λ (eV)[e]
CC/DTT-AuNCs	1.24	6.1	23	0.73	0.67
Met-AuNCs [2,3]	1.1	1.0	2.8	0.83	0.66
NAC-AuNCs [4,5]	0.75	0.77	1.2	1.04	0.66
MUA-AuNCs [6,7]	0.25	5.3	2.4	1.53	0.83
GSH-AuNCs [8,9]	0.80	1.4	15	0.93	0.67

[a] E_{ox} = oxidation potential (vs Ag/AgCl); [b] τ = luminescence lifetime; [c] QY = luminescence quantum yield; [d] ΔG = driving force; [e] λ = reorganization energy

Table S2. IFE on the luminescence response of CC/DTT-AuNCs toward AQ4N.

AQ4N (nM)	A_{ex}	A_{em}	CF	F_{obsd}	F_{cor}	$F_{cor,0}/F_{cor}$
0	0.288	0.000	1.37	592.2	809.0	1.00
25	0.298	0.000	1.38	544.0	750.9	1.08
50	0.281	0.000	1.36	511.2	693.6	1.17
75	0.291	0.000	1.37	483.5	662.7	1.22
100	0.284	0.000	1.36	453.7	617.4	1.31
125	0.282	0.000	1.36	426.0	578.4	1.40
150	0.291	0.000	1.37	406.8	557.5	1.45
175	0.299	0.000	1.38	395.3	546.3	1.48
200	0.289	0.000	1.37	360.5	493.1	1.64

References

1. Huang, K.; Fang, Q.; Sun, W.; He, S.; Yao, Q.; Xie, J.; Chen, W.; Deng, H, Cucurbit[n]uril Supramolecular Assemblies-Regulated Charge Transfer for Luminescence Switching of Gold Nanoclusters. *J. Phys. Chem. Lett.* **2022**, *13*, 419-426.
2. Xiu, L.F.; Huang, K.Y.; Zhu, C.T.; Zhang, Q.; Peng, H.P.; Xia, X.H.; Chen, W.; Deng, H.H. Rare-Earth Eu^{3+}/Gold Nanocluster Ensemble-Based Fluorescent Photoinduced Electron Transfer Sensor for Biomarker Dipicolinic Acid Detection. *Langmuir* **2021**, *37*, 949-956.
3. Deng, H.H.; Zhang, L.N.; He, S.B.; Liu, A.L.; Li, G.W.; Lin, X.H.; Xia, X.H.;Chen, W.

Methionine-Directed Fabrication of Gold Nanoclusters with Yellow Fluorescent Emission for Cu^{2+} Sensing. *Biosens. Bioelectron.* **2015**, *65*, 397-403.

4. Peng, H.; Jian, M.; Deng, H.; Wang, W.; Huang, Z.; Huang, K.; Liu, A.; Chen, W. Valence States Effect on Electrogenerated Chemiluminescence of Gold Nanocluster. *ACS Appl. Mater. Interfaces* **2017**, *9*, 14929-14934.
5. Deng, H.H.; Wu, G.W.; Zou, Z.Q.; Peng, H.P.; Liu, A.L.; Lin, X.H.; Xia, X.H.;Chen, W. pH-Sensitive Gold Nanoclusters: Preparation and Analytical Applications for Urea, Urease, and Urease Inhibitor Detection. *Chem. Commun.* **2015**, *51*, 7847-7850..
6. Sun, J.; Yue, Y.; Wang, P.; He, H.; Jin, Y. Facile and Rapid Synthesis of Water-Soluble Fluorescent Gold Nanoclusters for Sensitive and Selective Detection of Ag^{+}. *J. Mater. Chem. C* **2013**, *1*, 908-913.
7. Sun, J.; Wu, H.; Jin, Y. Synthesis of Thiolated Ag/Au Bimetallic Nanoclusters Exhibiting an Anti-Galvanic Reduction Mechanism and Composition-Dependent Fluorescence. *Nanoscale* **2014,** *6*, 5449-5457.
8. Tang, Y.; Xu, J.; Xiong, C.; Xiao, Y.; Zhang, X.; Wang,S. Enhanced Electrochemiluminescence of Gold Nanoclusters via Silver Doping and Their Application for Ultrasensitive Detection of Dopamine. *Analyst* **2019**, *144*, 2643-2648.
9. Luo, Z.; Yuan, X.; Yu, Y.; Zhang, Q.; Leong, D. T.; Lee, J. Y.; Xie, J. From Aggregation-Induced Emission of Au(I)–Thiolate Complexes to Ultrabright Au(0)@Au(I)–Thiolate Core–Shell Nanoclusters. *J. Am. Chem. Soc.* **2012**, *134*, 16662-16670.
